# Parametric Modeling and Optimization of Dimensional Error and Surface Roughness of Fused Deposition Modeling Printed Polyethylene Terephthalate Glycol Parts

**DOI:** 10.3390/polym15030546

**Published:** 2023-01-20

**Authors:** Prithu Mishra, Shruti Sood, Vipra Bharadwaj, Aryan Aggarwal, Pradeep Khanna

**Affiliations:** Department of Mechanical Engineering, Netaji Subhas University of Technology, New Delhi 110078, India

**Keywords:** PETG, FDM, dimensional accuracy, surface roughness, RSM, ANFIS, NSGA-II

## Abstract

Polyethylene Terephthalate Glycol (PETG) is a fused deposition modeling (FDM)-compatible material gaining popularity due to its high strength and durability, lower shrinkage with less warping, better recyclability and safer and easier printing. FDM, however, suffers from the drawbacks of limited dimensional accuracy and a poor surface finish. This study describes a first effort to identify printing settings that will overcome these limitations for PETG printing. It aims to understand the influence of print speed, layer thickness, extrusion temperature and raster width on the dimensional errors and surface finish of FDM-printed PETG parts and perform multi-objective parametric optimization to identify optimal settings for high-quality printing. The experiments were performed as per the central composite rotatable design and statistical models were developed using response surface methodology (RSM), whose adequacy was verified using the analysis of variance (ANOVA) technique. Adaptive neuro fuzzy inference system (ANFIS) models were also developed for response prediction, having a root mean square error of not more than 0.83. For the minimization of surface roughness and dimensional errors, multi-objective optimization using a hybrid RSM and NSGA-II algorithm suggested the following optimal input parameters: print speed = 50 mm/s, layer thickness = 0.1 mm, extrusion temperature = 230 °C and raster width = 0.6 mm. After experimental validation, the predictive performance of the ANFIS (mean percentage error of 9.33%) was found to be superior to that of RSM (mean percentage error of 12.31%).

## 1. Introduction

In recent years, additive manufacturing has grown rapidly to produce a variety of functional parts involving complex geometries with specific functionalities made from a wide range of materials. In contrast to traditional machining, where material is carved out from a block using suitable cutting tools to obtain the desired geometry [1,2], fused deposition modeling (FDM) is an extrusion-based AM process involving the layer-wise deposition of thermoplastic filaments in a semi-liquid state [3,4,5] using a computer-controlled deposition nozzle [6]. FDM allows researchers to obtain complex yet flexible and functional parts from a standard tessellation language (STL) file quickly, with high quality and a wide range of engineering thermoplastic materials [1], along with offering reduced assembly costs [7]. It is finding applications in the production of conceptual models/prototypes of functional parts for use in aerospace engineering [8], telecommunications [9] and automotive and biomedical implants. However, certain drawbacks, such as its lower dimensional accuracy, irregular surface finish and poor mechanical properties [7], limit its application. In recent years, numerous research endeavours have been performed that aimed to improve the quality and strength-related variables, including surface roughness, dimensional errors, strength and stiffness [10].

Dimensional accuracy is a measure of the variation in the size and specifications of the designed and the as-built parts [1,11] and improved dimensional accuracy is essential for ensuring a higher magnitude of stability and repeatability of the fabricated parts. For functional parts, dimensional accuracy is often accounted for by measuring the dimensional errors along different directions or features [7]. The dimensional errors introduced in the part have been found to depend on the printer parameters [11,12], part shape [13] and support material requirements. Surface roughness is another widely used index for assessing part quality and is often a technical requirement for mechanical products used in conjunction with customized product applications [14] such as automobile friction plates and sealing shafts. Good surface quality is important for reducing the overall lead time and improving the cost-effectiveness of the build process [15]. A pronounced staircase effect, a limited STL file resolution due to the limitations of the slicing procedure utilized and an improper selection of process parameters led to the FDM-built parts having a relatively poor surface finish.

The printing parameters affect the bonding between and within the deposited layers, and hence exercise a considerable influence on the production efficiency [7], energy consumption, manufacturing cost and time, and part build characteristics, such as the dimensional deviations, part finish and strength [1,16,17]. Hence, a study on the influence of these parameters and their optimization is of paramount importance while tackling the problem of reducing the dimensional errors and obtaining high surface quality.

Previous research has been performed to understand the influence of various FDM process parameters on dimensional accuracy in an attempt to minimize the dimensional deviations. Sood et al. [18] studied the influence of printing parameters on dimensional deviations along the length, width and thickness of acrylonitrile butadiene styrene (ABS) build parts based on Taguchi’s experimental design. The authors developed an artificial neural network model for the prediction of the deviations and further combined grey relational analysis and the Taguchi method to simultaneously minimize the dimensional deviations after expressing the three responses as a single response called the grey relational grade. Their results revealed that layer thickness was the most significant parameter affecting the width and thickness deviations; however, build orientation was the most influential on the deviation in the length. ABS-built parts were also studied for dimensional accuracy, build time and warp deformation by Peng et al. [19] with line width compensation, extrusion velocity, filling velocity and layer thickness as the input parameters. The authors converted the three output responses into a single comprehensive response (CR) using a fuzzy inference system (FIS) and then used response surface methodology (RSM) to develop models relating the CR to controllable factors. Their experiment revealed that lower layer thickness was desirable for improving dimensional accuracy. Alafaghani and Qattawi [20] studied the influence of infill percentage, infill pattern, layer thickness and extrusion temperature on the dimensional accuracy of poly-lactic acid (PLA)-based parts using Taguchi’s (L9) design of the experiments. They found better dimensional accuracy at a lower extrusion temperature and infill percentage, coupled with smaller layer thickness and hexagonal infill pattern. Tontowi et al. [21] compared the performance of the Taguchi orthogonal array and RSM in predicting the dimensional accuracy of PLA-based parts and concluded that RSM gave a better prediction. They observed that the raster orientation was more significant than the layer thickness for dimensional accuracy, unlike the other available literature. Alafaghani et al. [12] studied the impact of printing settings on mechanical properties, dimensional accuracy and repeatability of the FDM-built parts using PLA as the filament and considered four numerical parameters, including print speed, extrusion temperature, layer height and infill percentage, along with two categorical parameters: build orientation and infill pattern. They recommended lower values of the extrusion temperature and layer height for obtaining improved dimensional accuracy. 

Process optimization for reducing dimensional errors has also been performed for certain ABS blends and nylon. A PC-ABS blend made with a cuboidal-shaped part, built with the help of a soluble support material, was analysed for dimensional accuracy along with the length, width and thickness by Mohamed et al. [11]. They considered six process parameters, including the layer thickness, air gap, raster angle, build orientation, road width and number of contours, and used the I-optimal design approach to determine the optimal combination of process printing settings for the three-dimensional deviations. It was concluded that lower layer height and number of contours values led to reduced deviations across the three dimensions. Additionally, the other parameters were significant and affected the different dimensional deviations differently. An increase in air gap and road width along with a reduction in the raster angle reduced the dimensional deviations along the width and thickness but deteriorated dimensional accuracy along the length. Vishwas et al. [22] used the Taguchi (L9) orthogonal array and analysis of variance (ANOVA) for analysing the effects of varying the orientation angle, layer and shell thickness on the dimensional accuracy, ultimate tensile strength and manufacturing time of ABS and nylon-built parts. The optimal printing settings were different for the two materials, with layer thickness being the most significant input parameter, with a share of 84.84%, followed by shell thickness (12.66%).

ABS-printed parts were also analysed for surface roughness by Horvath et al. [23] using a full factorial experimental design taking model temperature, layer thickness and visible surface as input parameters. It was inferred that, while model temperature was insignificant, layer thickness had a higher influence on surface roughness. Their study also suggested that a finer raster width was beneficial for improving the surface finish. A full factorial experimental plan was employed by Galantucci et al. [24] to study the influence of process parameters, namely, tip dimension, raster width and slice height, on the surface roughness of ABS-built parts. It was observed that slice height and raster width were important factors; the tip diameter, however, had little significance for surfaces running either parallel or perpendicular to the build direction. Saad et al. [14] used RSM to develop a regression model to establish a relationship between surface roughness and the input responses, including layer height, print speed, print temperature and outer shell speed. The group then coupled the model with particle swarm optimization and symbiotic organism search to optimize the input parameters. The experimental results highlighted an improvement of 8.5% and 8.8% in the obtained part finish using particle swarm optimization and symbiotic organism search, respectively, as compared to RSM. The authors concluded that metaheuristic methods can improve the FDM part finish. Experiments have been performed involving the multi-objective optimization of surface roughness and other important measurable parameters for ABS-built parts. Thrimurthulu et al. [25] solved the problem of simultaneously optimizing the part surface finish while reducing the build time using a genetic algorithm. The study recommended a lower layer thickness for a high surface finish and it was revealed that the developed model could be utilized to anticipate the optimal part orientation for any complex freeform surface. Another work aimed at improving the surface roughness, hardness, tensile strength and flexural modulus of ABS-built parts was performed by Raju et al. [26] using the Taguchi (L18) orthogonal array. Different empirical models were developed based on linear multiple regression and ANOVA analysis for studying the relationships of the responses with parameters including layer thickness, support material, model interior and build orientation. It was concluded that layer thickness and build orientation were two significant parameters for surface quality. The multi-objective optimization of the said responses was implemented using particle swarm optimization, bacterial foraging optimization and a hybrid of the two algorithms.

Similar to ABS, PLA-built parts have also received attention with regards to improving the obtained part finish. Peng and Yan [27] attempted to characterize the surface roughness and energy consumption of PLA parts during printing in their study based on a full factorial design involving layer thickness, infill ratio and the printing speed as the input parameters. It was observed that layer thickness was a contradictory parameter amongst the two output responses. Smaller layer thickness was found to give a smoother surface finish, aligning with the study by Perez et al. [28] in which the influence of layer thickness, shell thickness, extrusion temperature and print speed on the surface roughness of PLA-built cylindrical-shaped specimens was analysed using graphical and statistical tools including ANOVA, Spearman’s ρ and Kendall’s τ correlation coefficient. The surface roughness and tensile strength of PLA parts were studied by Altan et al. [29] in a study based on the Taguchi (L16) orthogonal array with layer thickness, deposition head velocity, nozzle temperature and cooling effect on the samples as the input parameters. ANOVA analysis revealed the former three parameters to be significant, with the effect of layer thickness being similar to the previous work. Nozzle temperature or the use of cooling fans were found to be the least effective parameters for controlling surface roughness.

Apart from these works, attempts focussing on the multi-objective optimization of the two output responses of interest were also reviewed. Anusree et al. [30] conducted a study investigating the influence of layer thickness, raster width, print speed and support material density on the dimensional accuracy, tensile strength and surface finish of ABS build helical parts. The study was unique, as the surfaces selected for experiment were helical instead of flat or circular, as opted for in the majority of the works in this field. Similar to Sood et al. [18], the three responses were converted into grey relational grade, which was further maximized using the Taguchi method. For the optimal parameter configuration, a smaller layer thickness value, higher raster width and intermediate print speed in conjunction with a rough support material density was recommended. The same three output responses were considered by Chung Wang et al. [31] with the input parameters being layer thickness, deposition style, support style, deposition orientation in the Z and X direction, and build location for ABS-built parts. The experimental methodology was similar to Anusree et al. [30], but with an extension of the verification of the results using a technique for ordering performance by similarity to the ideal solution. The findings of the study based on ANOVA revealed that deposition orientation in the Z direction and layer thickness were the most influential factors on dimensional accuracy and surface roughness, respectively. Bakar et al. [32] studied the variation in dimensional error and surface roughness with changing part shapes by considering different shapes, including slot, cube, ring and cylinder. The dimensional accuracy and surface roughness were found to depend on the specimen shape, with the cylindrical shape generally leading to higher dimensional deviation, whereas a complex curvy surface was found to give a deteriorated surface finish. A wider raster width was recommended for an improved surface finish while layer thickness was found to have a similar influence as reported in the reviewed literature. 

It can be inferred from the literature review that the problem of minimizing the dimensional errors and surface roughness of FDM-built parts using parametric optimization is well known and studied by numerous research groups. For dimensional accuracy, lower levels of layer thickness and extrusion temperature are desired. Out of the several input parameters considered, the effects of varying the raster width and print speed have not been comprehensively reviewed and understood. For surface roughness, layer thickness has been determined to be the most significant parameter, followed by raster width, with lower levels of both recommended for obtaining an improved surface finish. Print speed is also a significant parameter influencing the surface finish of the built part. Moreover, the dimensional deviations were found to have different behaviour along the length, width and height of the samples and a different combination of parameters is desirable for minimizing these deviations. The dependence of surface roughness on the specimen shape has also been highlighted, with its magnitude being influenced by the flat or curvy nature of surfaces, as well as their orientation, i.e., flat or vertical. 

Therefore, in the present work, the influence of selected input parameters on the dimensional deviations along the length, width and thickness, along with the surface finish of both flat as well as complex curvy surfaces, was investigated with an aim to achieve combinations of parameters that would lead to high dimensional accuracy and part finish for varying directions and differently oriented surfaces. Also, as both of these quality objectives are often concurrently desired and are differently influenced by different parameters [10], a need for the simultaneous optimization of the objectives was felt. Since the FDM process involves numerous conflicting parameters, the conventional optimization methods, such as the Taguchi and grey relational analysis [1] which have been used by majority of researchers, are less suitable due to their limited proficiency in describing a complex functional relationship for a non-linear process involving a number of interacting parameters [18,33]. The present investigation hence utilizes the non-dominated sorting genetic algorithm II (NSGA-II), an evolutionary algorithm that generates a set of non-dominated solutions called the pareto front and allows the user to select the most fit solution from a given set of solutions.

It is inferred that most of the research work undertaken in the field of parametric optimization for the improvement of mechanical and physical parameters of FDM-built parts is dedicated to ABS and PLA material. In recent times, attempts have been made to investigate the applicability of previously unexplored FDM materials for various applications. Rahmatabadi et al. [34] explored the mechanical properties of food-grade, unmodified polyvinyl chloride for potential biomedical applications. The authors considered numerous tension modes, including compression, bending and tension, and studied the variation in strength with varying printing parameters. The study revealed the raster angle and print speed to be significant parameters, while the layer thickness and nozzle diameter had little effect on the said responses. Another FDM-compatible material, polyethylene terephthalate glycol (PETG), is gaining popularity in recent times. PETG is a glycol-modified version of polyethylene terephthalate (PET) [35] having high toughness, chemical resistance, durability, low forming temperature and, hence, proving to be a good material for thermoforming [36], extrusion, injection molding and FDM [36,37,38,39,40]. In recent times, PETG has found applications in medical industries, food packaging industries, electronics, etc. [41,42]. In FDM, the important benefits of printing with PETG over ABS include higher durability and strength, lower shrinkage with little or no warping, better recyclability [43], lower particle and volatile organic compound (VOC) emissions, and improved chemical resistance to alkali, acid and water [44]. Expanding on its application, Soleyman et al. [45] investigated the 4D printing capability and shape memory effect of PETG as a novel shape memory thermoplastic. The authors studied the appearance of a curved third shape sample as a function of printing temperature and speed and observed pronounced shape memory-affecting behaviour, with the total shape recovery exceeding 96%. These advantages have led researchers to perform investigative works to explore the potential of PETG as an alternative to ABS and PLA, as a filament material [46]. However, the majority of research efforts dedicated to the FDM printing of PETG parts aimed to analyse the relation between the process parameters and mechanical behaviour of the test specimens [47,48,49,50]. An eminent research gap was felt in studies aiming to understand the influence of FDM process parameters on dimensional errors in different directions and surface roughness on varied part surfaces and features along with the subsequent parametric optimization of the parameters. Additionally, no work has been reported performing the multi-objective optimization of PETG parts to minimize dimensional deviations while simultaneously obtaining improved part finishes. 

The present research work aims at studying the effect of FDM process parameters, namely, print speed, layer thickness, extrusion temperature and raster width, on the dimensional accuracy and surface roughness of FDM-printed PETG parts. Since dimensional errors along the three directions behave differently, this work aims to understand these variations and how these are governed with the varying printing parameters. As an extension, the influence of the parameters on the dimensional errors introduced in a hole incorporated in the geometry is also considered. In the study, the specimen geometry and shape were selected to allow for the analysis of the significant parameters and their influence on the surface roughness of flat, inclined and curved surfaces. A four-factor, five-level central composite rotatable design was employed for performing a series of experiments. The recorded experimental data were statistically modelled using RSM and the adequacy of the developed models was verified using ANOVA. The significant parameters and their manner of influence on each of the seven output responses, including four dimensional deviations (X, Y, Z directions and hole) and three surface roughness responses (flat, inclined and curved), were considered. A Sugeno-type ANFIS model was also developed for the prediction of output response parameters and multi-objective optimization was conducted using an integrated RSM and NSGA-II algorithm approach. A phase-wise multi-objective optimization was performed to identify the combination of the input parameters that minimizes the dimensional errors as a whole, the combination that improves the overall surface finish and finally the combination that yields a parameter setting that will enable the printing of complicated PETG parts with the overall best quality. To validate the obtained results and compare the predictive performance of the developed models, the required validatory experiments were performed.

## 2. Materials and Methods

### 2.1. Experimental Plan and Procedure

**Figure 1:**
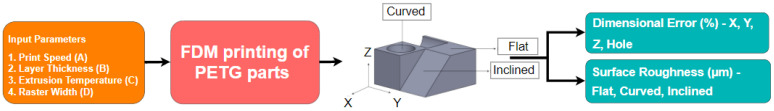
The selected input and output parameters for the FDM printing of PETG parts.

Different process parameters influence the induced dimensional errors and the obtained surface finish on FDM-built parts to a lesser or greater extent [7,11,51,52,53]. In the present study, the input parameters selected for assessing the dimensional accuracy and surface roughness included print speed (A), layer thickness (B), extrusion temperature (C) and raster width (D), as shown in Figure 1. The printing parameters can be briefly defined as follows: 

Print Speed is referred to the travel speed of the print head along the XY plane (parallel to the build platform) while extruding.

Layer Thickness is the height (or thickness) of layers deposited after extrusion from nozzle tip measured along Z-direction (perpendicular to the build platform).

Extrusion Temperature is referred to as the temperature at which the filament material is heated in the liquefier before extrusion [52]. It depends on properties of thermoplastic material being used. 

Raster Width is the width of the molten filament which is deposited on the FDM printer bed [54]. It depends on the diameter of the extruder nozzle tip.

The working ranges of the four selected process parameters were divided into five levels, as shown in Table 1. They were chosen on the basis of literature survey, hardware and printer specification restrictions, Ultimaker Cura 15.04.6 building simulation and trial experiments. The trial experiments were conducted in such a manner that only one of the FDM process variables was varied keeping the rest of them fixed. The upper and lower limits were decided based on part quality obtained between them.

Based on the process parameters and their working levels, design of experiment’s technique was employed to generate a design matrix using Design Expert v13. To develop the empirical models for dimensional accuracy and surface roughness, four-factor central composite technique of rotatable configuration was employed, consisting of a total of 30 runs. The 30 runs comprised of $2^{4}=16\;$ runs using half factorial, 6 runs representing center points and 2 × 4 = 8 runs representing star points [55].

**Figure 2:**
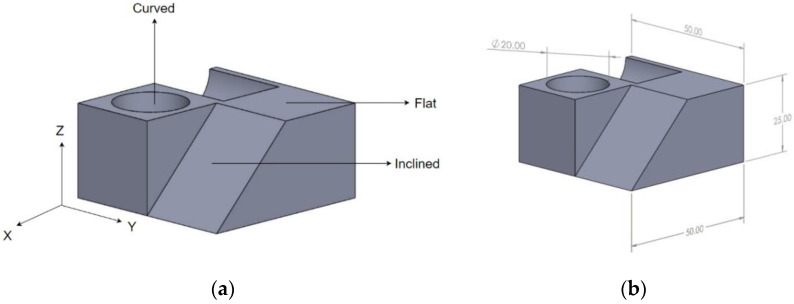
Designed part highlighting the (**a**) dimensions (mm) and (**b**) surface profiles.

With an aim of understanding the distinct manner of influence of the process parameters and arriving at parameter settings to enable optimal printing of complex FDM-printed PETG parts, the specimen geometry was designed such that it had varying surfaces and contours to measure the desired responses. The part was designed using SolidWorks 2021 and had a dimension of 50 × 50 × 25 mm with a hole of 20 mm diameter as shown in Figure 2a. The dimensional errors were measured along the three cartesian axes (X, Y and Z direction) to understand the extent of influence of the dynamic effect of the nozzle (controlling the X and Y direction) and the printer bed drive system (controlling the Z direction), along with the tendency of material expansion and shrinkage across different directions. The hole geometry was studied to further investigate the proneness of material spreading when unconstrained by surrounding extruded filament as a function of varying printing settings. Since the nature of material deposition varies for different surface profiles and features, the printing parameters influence the surface finish of these surfaces differently, as also observed by Khan and Mishra [56]. In line with the objective of the study, the specimen geometry incorporated flat, inclined and curved surfaces (Figure 2b), to enable the recording and analysis of effects of selected printing parameters on the surface roughness of the as printed profiles.

The STL file of the CAD drawing was loaded into Ultimaker Cura to slice the model file into layers, set process parameters and generate a printer-specific g-code for each specimen. The parts were printed as per the instruction code using ShaperJet SJ200 based on FDM technology, having a build size of 200 × 200 × 200 mm, printing speed ranging from 10 to 100 mm/s, extruder temperature 180–270 °C and a blower for cooling printed parts. All the samples were prepared with the raster angle of 45° and printer bed temperature fixed at 70 °C to ensure sufficient bonding of the first layer and avoid warpage of samples. In order to vary the raster width, five different diameter nozzles (0.2 mm, 0.3 mm, 0.4 mm, 0.5 mm, and 0.6 mm) were used. 3DXTech PETG filament wire having a diameter of 1.75 ± 0.05 mm and density of 1.24 g/cm^3^ was employed for the experiment.

**Figure 3:**
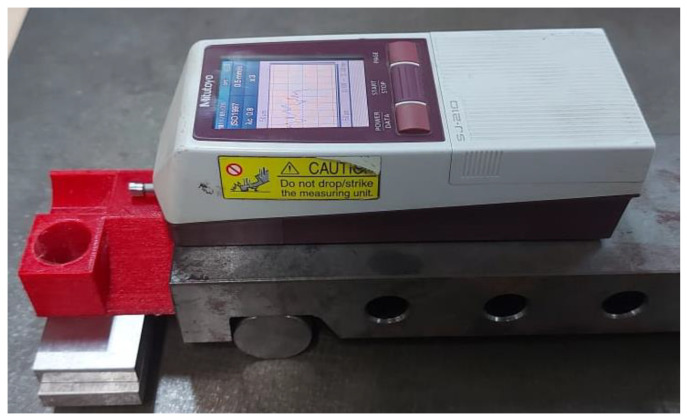
Surface roughness measurement using SURFTEST SJ-210.

**Figure 4:**
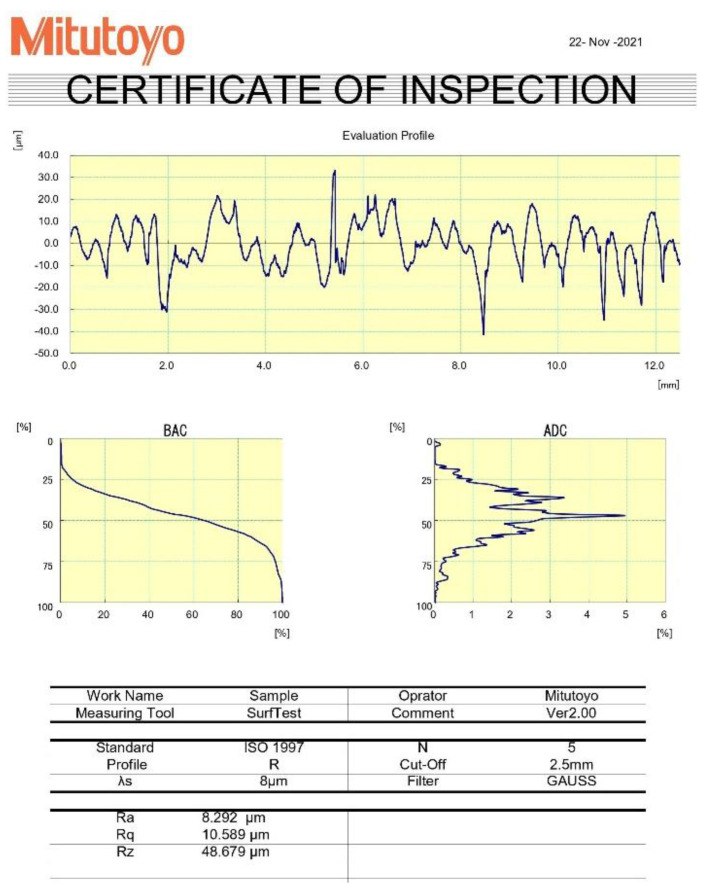
Surface roughness report generated by SURFTEST SJ-210 for run 12.

Once the parts were printed, the dimensional error and surface roughness measurement was done. To measure the dimensional error along the cartesian axis (X, Y and Z) and cylindrical hole, a Mitutoyo manufactured vernier calliper having a least count of 0.02 mm was used. Three readings of dimensional error were taken along each of the cartesian axes and four readings were taken for the dimensional error of the hole. The mean values of the recorded responses are as shown in Table 2. For surface roughness measurement of the flat, inclined and curved profiles, SURFTEST SJ-210, a compact and portable surface roughness tester, manufactured by Mitutoyo with the lowest count of 0.001 µm was used. The stylus moves across the specimen surface with defined measuring speed and length to trace the irregularities on the workpiece surface. The surface profile and surface roughness (R_a_) are displayed as the output on the screen. For the purpose of this measurement, a Gaussian filter was applied and ISO 1997 roughness standard was followed. The cut off wavelength was set at 0.25 mm, measuring speed at 0.5 mm/s and number of sampling lengths at x5. The direction of surface roughness measurement was perpendicular to the lay direction and a sine bar was also used along with SJ-210 for measurement of inclined and curved profiles. The setup used for measurement is shown in Figure 3 while, Figure 4 shows a sample report for flat profile generated by SJ-210 for run 12. A total of five readings were taken for the flat profile and four readings each for inclined and curved surfaces, and their mean values are as shown in Table 2. The readings as shown in Table 2, were supplied to the design of experiments and were used as the input data for making the predictive models as explained further. 

### 2.2. Statistical Modelling of Experimental Data

Response surface methodology (RSM) was used to develop statistical models for different output parameters owing to its better predictive performance for low order non-linear processes with a regular experimental domain, as reported by Benyounis et al. [55]. The factor contributions derived from the coefficients in the RSM developed regression model allow for identifying the insignificant factors and interactions, thereby reducing the complexity of the problem. In this study, Design Expert v13 software was used to develop second order quadratic RSM models (shown in Equation (1)) on the input data given in Table 2 to estimate linear, interaction and quadratic effects of the input factors and to provide prediction models for response parameters.

Let Q denote the predicted response value dependent on the four input parameters namely, print speed (A), layer thickness (B), extrusion temperature (C) and raster width (D). Then, Q can be written as

(1) $$Q=\gamma_{0}+\sum \gamma_{i}p_{i\;}+\sum \gamma_{ii}p_{i}^{2}+\sum \gamma_{ij}p_{i}p_{j}$$

where Q = predicted response value, $p_{i},\;p_{j}$ = coded values of the input parameters (A, B, C, D), $\gamma_{0}$ = regression equation constant, $\gamma_{i}$ = linear coefficient, $\gamma_{ii}$ = square term of each parameter, $\gamma_{ij}$ = first order interaction effect.

The regression equations for the response parameters are as given in Equations (2)–(8).

(2) $$\begin{array}{ll} Dimensional & Error\;X-DE_{x}\;\left(\%\right)\\ & =0.7733+0.0994\times A+0.0783\times B+0.0789\times C+0.0850\times D+0.0475\times AB+0.0175\times AC\\ & +0.0242\times AD+0.0392\times BC+0.0325\times BD+0.0258\times CD-0.0108\times A^{2}-0.0542\times B^{2}\\ & -0.01\times C^{2}-0.0508\times D^{2} \end{array}$$

(3) $$\begin{array}{ll} Dimensional & Error\;Y-DE_{y}\;\left(\%\right)\\ & =0.411+0.0430\times A+0.0744\times B+0.0369\times C+0.0544\times D+0.0716\times AB-0.0622\times AD\\ & -0.0477\times BD-0.0197\times A^{2}-0.0305\times B^{2}-0.0175\times C^{2}-0.0464\times D^{2} \end{array}$$

(4) $$\begin{array}{ll} Dimensional & Error\;Z-DE_{z}\;\left(\%\right)\\ & =0.3674+0.0704\times A+0.1319\times B+0.0819\times D+0.0806\times AC+0.0578\times AD+0.0761\times BC\\ & +0.0322\times BD-0.0389\times CD+0.0471\times B^{2}+0.0438\times D^{2} \end{array}$$

(5) $$\begin{array}{ll} Dimensional & Error\;Hole-DE_{hole}\;\left(\%\right)\\ & =2.21+0.0480\times A+0.0835\times B+0.0656\times C+0.1498\times D-0.0434\times AB+0.0516\times AC\\ & +0.0303\times AD+0.0153\times CD-0.0426\times B^{2}\\ & -0.1594\times D^{2} \end{array}$$

(6) $$\begin{array}{ll} Surface\;Roughness & Flat-SR_{flat}\;\left(\mu m\right)\\ & =8.09-0.0137\times A+2.24\times B+0.0139\times C+0.2002\times D-1.58\times AB-1.02\times AC\\ & +0.7731\times AD+0.5870\times BC-0.3177\times BD+0.4464\times CD+0.4422\times A^{2}+0.2381\times B^{2}\\ & +1.21\times C^{2}-0.1817\times D^{2} \end{array}$$

(7) $$\begin{array}{ll} Surface\;Roughness & Inclined-SR_{inclined}\;\left(\mu m\right)\\ & =28.17+0.3136\times A+3.87\times B-0.3499\times C+0.4374\times D+0.3656\times AB-0.4282\times AC\\ & -0.7328\times AD-0.1920\times BC+1.04\times BD-0.1590\times CD-0.1519\times A^{2}-0.6186\times B^{2}\\ & +0.3524\times C^{2}-0.1677\times D^{2} \end{array}$$

(8) $$\begin{array}{ll} Surface\;Roughness & Curved-SR_{curved}\;\left(\mu m\right)\\ & =22.29-0.0541\times A+1.58\times B-0.4925\times C+1.90\times D+0.7695\times AB-0.7225\times AC\\ & +0.5420\times AD-0.7238\times BC+0.7638\times BD+0.1435\times CD-0.5248\times A^{2}-0.4236\times B^{2}\\ & +1.18\times C^{2}-0.0682\times D^{2} \end{array}$$

The appropriacy of the models developed was confirmed using analysis of variance (ANOVA) and the values obtained are as shown in Table 3. ANOVA was used to test the significance of the developed models and identify the significant linear, quadratic and interaction terms for all output responses. The details on working of ANOVA can be found in Kaufmann et al. [57]. The *p*-value for developed models of all responses was found to be <0.05, which implied that the models were significant at 95% confidence level. The terms with *p*-value lower than 0.05 were considered significant and are mentioned in Table 4. The high R^2^ values as given in Table 3 are desirable as it shows that a high percent of the observed variation of the dependent variable can be explained based on the alterations in the independent input printing parameters. Higher R^2^ values denote lesser influence of external noise on the developed models. Adeq Precision is a measure of the signal-to-noise ratio of the developed models and values greater than 4 verify the signal adequacy. 

### 2.3. ANFIS Model for Prediction 

Adaptive neuro fuzzy inference system (ANFIS) refers to a neuro-fuzzy system inheriting the advantages of both FIS network and artificial neural network by developing a multilayer feed forward neural network [58]. An artificial neural network can enhance the learning process but has limitation with regards to deduction and expression of the knowledge acquired. The FIS on the other hand, has good knowledge learning capabilities based on linguistic variables that are easy to comprehend and follow, but lacks the learning capabilities of artificial neural network [59]. An ANFIS network integrates these advantages and overcomes the individual drawbacks by employing the neural network architecture for adjusting the FIS parameters including the membership functions and the IF-THEN rules [60]. This working approach renders ANFIS effective in a broader range of real-world applications including FDM process performance prediction [59].

**Figure 5:**
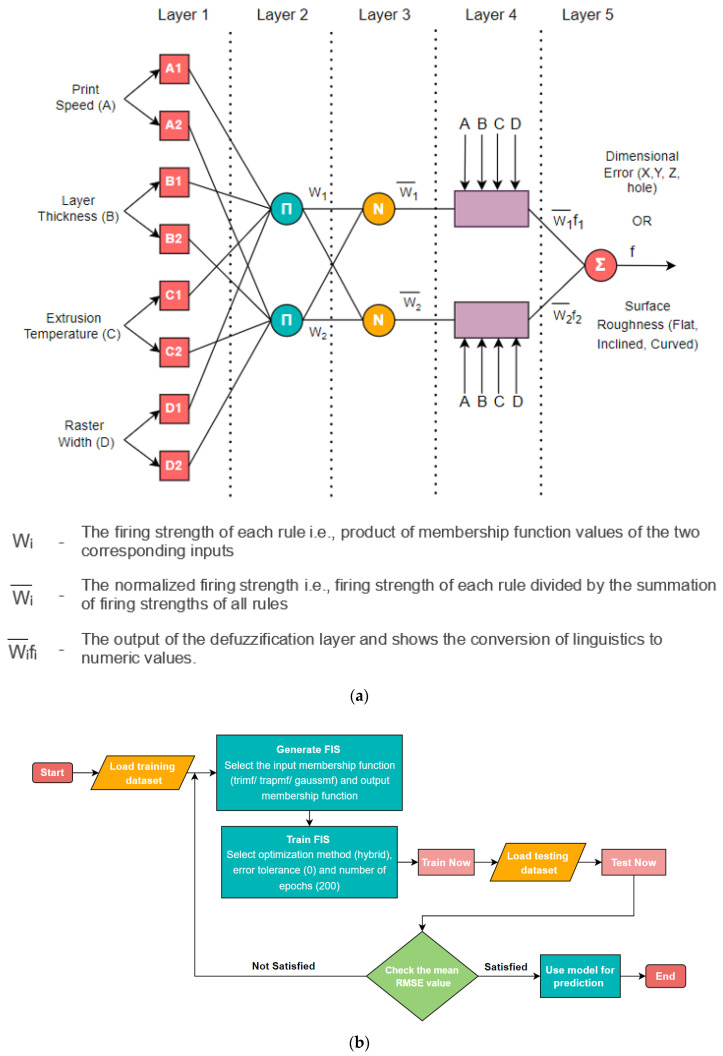

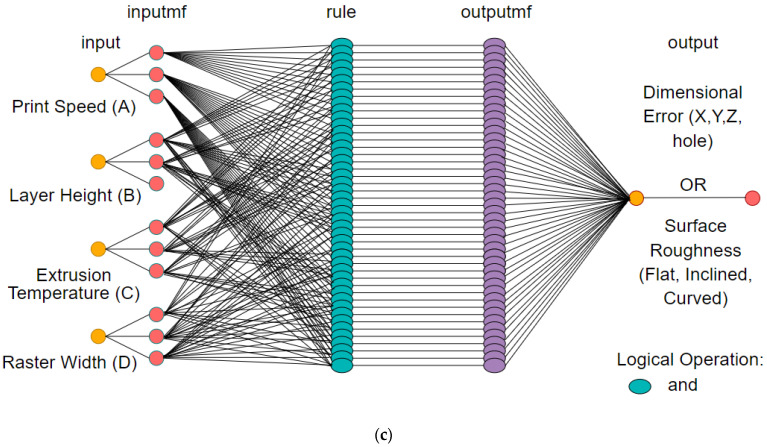
(**a**) ANFIS structure (**b**) ANFIS workflow (**c**) representative ANFIS architecture generated in MATLAB.

An ANFIS network involves five network layers along with a learning algorithm. The learning algorithm modifies the model as per the input and output dataset by adapting the parameters including, the membership functions and the firing strength of the rule. A brief working of the ANFIS architecture is described here, however, for deeper understanding one can refer Rajpurohit and Dave [61]. Layer 1 as shown in Figure 5a, is the fuzzification layer wherein, each input node is an adaptive node which is converted into linguistics using the membership functions. In the product layer i.e., layer 2, each node is fixed and gets multiplied by the incoming signals, and the firing strength of the rule is adjudged. Normalization of the firing strength of each node from layer 2 is performed in layer 3 where all the nodes are fixed. Layer 4 is the adaptive defuzzification layer where the normalized firing strength of each rule is multiplied with the resulting output response. Layer 5 is the final layer that yields the output modelled by ANFIS network.

In the present study, a Sugeno type ANFIS model was developed consisting of four inputs (A, B, C, D) for each response variable. Moreover, the network used the hybrid learning algorithm which employs the least square method in the forward passage to identify the network parameters and the gradient descendent method while propagating backwards. The hybrid learning approach is very efficient as it converges much faster compared to back propagation algorithm and avoids the tendency of getting trapped at a local minimum [58,60,61].

The Neuro Fuzzy Design tool of MATLAB MathWorks was used to model ANFIS structure for predicting the values of the four responses for dimensional error and the three responses accounting for the surface roughness for different surface profiles. The ANFIS modelling workflow is summarized in Figure 5b.

The ANFIS network is developed by training the algorithm on a training dataset containing the input/output pairs needed to form the network. The supplied inputs and outputs in the training data set are adaptively mapped using different membership functions, rule bases, and associated parameters acquired from the loaded learning dataset. From the obtained experimental data approximately 85% of the observations were utilized for training the model and finding the premise parameters for the membership functions while the remainder data was reserved for testing purposes. After the training data was loaded in the neuro-fuzzy designer module, the next step involved the generation of FIS which entailed the selection of the membership function. For the study, three different membership functions, triangular membership function (trimf), trapezoidal membership function (trapmf) and gauss membership function (gaussmf) were used to develop three different ANFIS models for each response parameter. 

Further, the epochs value was set at 200 and the hybrid optimization method was selected as the learning algorithm with zero error tolerance. The model was then trained with the specified parameters and upon completion of training, dataset for testing was loaded in the ANFIS system. Subsequent testing was carried out for all three membership functions and the root mean square error (RMSE) was recorded for each test performed. Finally, the model with the least average testing error was selected as the final model for prediction purpose. Table 5 documents the average testing error for each of the three selected membership functions for the different quality responses. The bold value highlights least value of RMSE and hence, the model selected for response prediction. 

**Figure 6:**
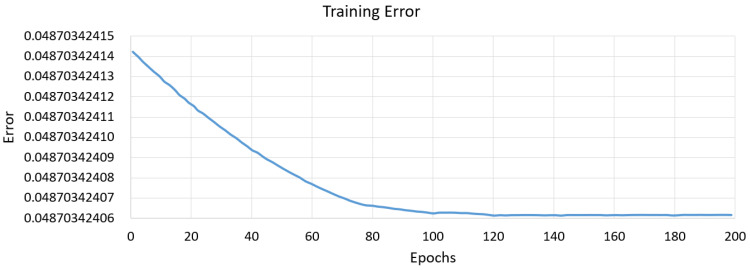
Training Error in dimensional Error X.

**Figure 7:**
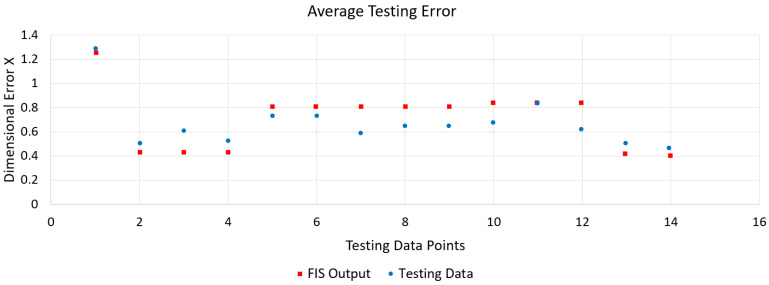
Average testing error for the ANFIS model for dimensional error X.

Figure 5c shows a representative ANFIS architecture as modelled in MATLAB. The dynamic training error behaviour against the training dataset during the training of the ANFIS model for dimensional error in X direction based on the trapezoidal membership function is shown in Figure 6. After 200 epochs the error converged to a constant value of 0.048703 signifying adequate model training. Figure 7 gives the variation in the values of dimensional error in X obtained after testing (red asterisks) from the original testing data point values (blue data points) and it was observed that the maximum % error was less than 0.4%. The low errors for all the seven quality responses confirmed that the values predicted by the developed ANFIS models were in good proximity with the experimentally measured values.

### 2.4. Multi-Objective Optimization Using Integration of RSM and NSGA-II

Multi-objective optimization of the response parameters was performed by integration of RSM and NSGA-II. NSGA-II, a widely used multi-objective evolutionary algorithm is an improved version of NSGA and overcomes limitations such as computational complexity, lack of elitism, and selection of optimal parameter value for sharing parameter [62]. NSGA-II applies binary tournament selection, elitist preserving strategy, non-dominated sorting, and crowding distance mechanism to obtain a good quality and uniformly spread non-dominated solution set [63]. The algorithm begins by randomly generating an initial population which is sorted based on non-domination, into fronts. Every individual in each front is assigned rank (fitness) values based on the front it belongs to. In addition to this, crowding distance which is a measure of how close an individual is to its neighbours is also calculated. The parents for the next generation are selected using binary tournament based on rank and crowding distance. An individual with lower rank is preferred during selection, however, in case the rank is same for the two individuals, higher crowding distance is used as the selection criteria. To produce the new population, genetic operators are applied and both the populations, initial and newly formed, are combined to perform non-dominated sorting. The best individuals equal to the population size are then selected and for the last front, selection is again based on the rank and crowding distance [64].

In the present work, multi-objective optimization was performed in three phases to identify optimal parameter combinations for reducing all the dimensional errors (DE_x_, DE_y_, DE_z_, DE_hole_), for minimization of surface roughness for different surface profiles (SR_flat_, SR_inclined_ and SR_curved_) and for simultaneously minimizing the dimensional errors and obtaining the best overall surface finish. The ‘gamultiobj’ algorithm in MATLAB’s optimization toolbox was used whose elitism is controlled by two options, ParetoFraction and DistanceMeasureFcn. The former option limits the elite members, i.e., the number of members on the pareto front and the latter maintains the diversity of the population by preferring the members which are relatively far away on the front. To perform the multi-objective optimization, quadratic expressions furnished by RSM, expressed in Equations (2)–(8) were used as the objective functions in the algorithm. The constraints for the optimization problems included lower and upper bound of input parameters, non-negative constraints and the upper bounds for the responses based on the obtained experimental data.

**Figure 8:**
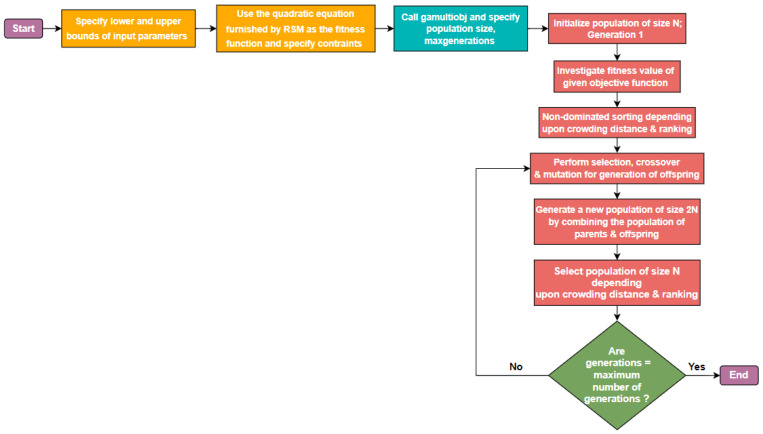
Flowchart for the hybrid RSM and NSGA-II approach.

For all three multi-objective optimization problems, the ‘gamultiobj’ options were kept uniform. The population size was defined as 500, the maximum generations was set at 200, crossover fraction was fixed at 0.8 and elite count was taken to be 0.05 of population size. The algorithm terminated with the message “Optimization terminated: average change in the spread of Pareto solutions less than options. FunctionTolerance.” highlighting that the relative improvement in the generated pareto solutions for successive iterations was lower than the minimum threshold as specified in FunctionTolerance which was set at the default value of 1 × 10^−4^. The “gamultiobj” algorithm returned a set of 175 non-dominated or pareto solutions for each problem. Figure 8 summarizes the detailed workflow used in the study for integrated RSM and NSGA-II approach.

**Figure 9:**
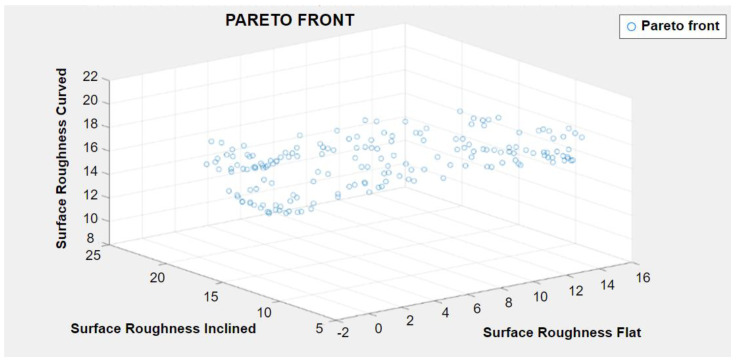
The generated pareto front for minimizing the surface roughness.

The solutions generated by NSGA-II are called non-dominated as they are all equally optimal and there is no single solution that simultaneously optimizes all objectives [65,66]. For extracting a single solution, an additional decision-making process was implemented keeping in view the optimization goals along with making necessary trade-offs. The best solutions for the multi-objective optimization problems as recommended by the hybrid approach are summarized in Table 6. Figure 9 shows the graphical depiction of the pareto front obtained for multi-objective optimization problem for simultaneous minimization of surface roughness of all profiles.

In practice, the printing parameters can only take certain discrete values at constant steps in almost all commercially available FDM printers due to limitations of the printer hardware and layer tessellation precision. Therefore, the optimal values of input parameters as suggested by the algorithm were shifted to the nearest coded level for ensuring universal applicability of results. The shifted readings are as summarized in the Table 7.

## 3. Results and Discussion

### 3.1. Discussion on Dimensional Error

**Figure 10:**
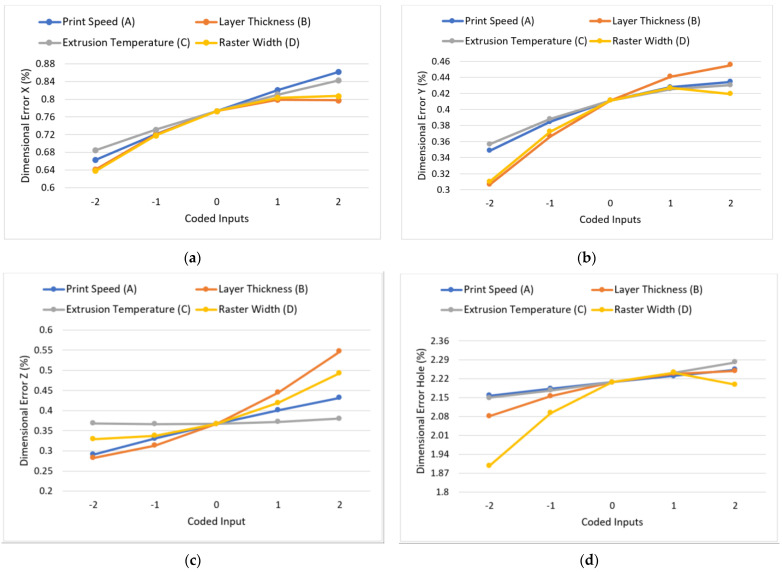
Effect of variation of input parameters on (**a**) dimensional error in X (%), (**b**) dimensional error in Y (%), (**c**) dimensional error in Z (%) and (**d**) dimensional error in hole (%).

Figure 10a–d depicts the response plots of dimensional errors DE_x_, DE_y_, DE_z_ and DE_hole_, showing the effect of various process parameters. ANOVA revealed the layer thickness and raster width to be significant parameters for all the dimensional deviations, which is further supported by the responses’ noticeable fluctuation with varying levels of the said parameters. In the FDM process, the heat carried by the extruded material is dissipated via modes of conduction and forced convection as the material cools down from the extrusion temperature to the glass transition temperature. The temperature reduction, as a consequence of this phenomenon, forces the material to rapidly solidify onto the previously deposited layers. The heat content of the just-extruded material, however, causes the localized remelting of the previously solidified material, leading to bonding amongst the deposited rasters [18]. This sequence of rapid cooling and heating cycles results in setting up non-uniform thermal gradients within the deposited material and in turn amplifying its tendency to undergo dimensional distortion or warping. The warping behaviour is restricted as the number of layers is increased, as observed by Wang et al. [67], owing to the lower thermal stress accumulation [11]. A similar trend was observed in the present study, wherein the percentage increase in dimensional errors was reported to be 24%, 49%, 93% and 11% for DE_x_, DE_y_, DE_z_ and DE_hole_ as layer thickness increased from 0.1 mm to 0.3 mm. At lower layer thickness values, the number of layers deposited for part fabrication increased and, as a consequence, the dimensional errors were minimized. The high increase in DE_z_ can be attributed to the height error observed along the Z direction, as was also observed by Mohamed et al. [11]. The dimension of the designed part was 25 mm in the Z direction, resulting in the number of layers required to be deposited for printing being 250 and 83.33, respectively, as the layer thickness varied from 0.1 mm to 0.3 mm. In the latter case, 83 layers were deposited normally; however, to account for the left 0.33 layers, the printer deposited another layer (84th) at the layer height of 0.3 mm. Hence, there was an increase in the fabricated part height with reference to the designed height, leading to more pronounced dimensional deviations in the Z direction at a higher layer thickness. 

As observed in Figure 10a–d, the dimensional errors reduced as the raster width was reduced from 0.6 mm to 0.2 mm. At finer raster widths, the material exiting the nozzle was reduced, hence the heat content entering the system environment was also less. The lower heat content restricted the localized remelting of the previously deposited material, thereby limiting the thermal stress accumulation at the interface [18]. Moreover, a precise raster width inhibited the radial expansion of the polymer melt exiting the nozzle [54] and hence led to reduced dimensional errors.

For dimensional errors DE_x_, DE_y_ and DE_z_, print speed was another significant parameter, as also summarized in Table 4. The increased dynamic effect of the nozzle drive system at higher print speeds gave rise to a jerky motion [52] of the nozzle. The jerky movement inhibited the ability of the nozzle to deposit precise rasters and hence contributed to high dimensional errors in the XY plane. An increase of 27% and 35% was noted for DE_x_ and DE_y_ as the print speed increased from 20 mm/s to 80 mm/s. The percentage increase, however, was found to be even higher in DE_z_ (approximately 50%), due to the combined action of the dynamic drive effect and the drawing phenomenon [19]. The latter occurs at high print speeds, wherein the extruded filament does not adhere to previously fabricated layers but rather moves with the nozzle due to there being insufficient time available for fusing with deposited layers. Thus, for improved dimensional accuracy, lower print speeds are recommended.

The extrusion temperature was found to exercise a significant influence on DE_x_ and DE_y_. The increased material fluidity at higher extrusion temperatures led to excessive material spreading post-deposition [12], posing another challenge for depositing precise rasters. However, in the Z direction, the magnitude of the error remained nearly constant, with varying extrusion temperature highlighting that the increased material fluidity exerted a lesser pronounced effect in the Z-direction as compared to the XY movement plane of the printer.

Moreover, the magnitudes of dimensional errors introduced for the internal feature, i.e., the curved hole surface, were, on an average, four times higher than the dimensional errors along the X, Y and Z directions. The explanation for the same can be based on the higher tendency of the semi-molten material to spread, especially when unconstrained by the surrounding material, as in the case of the hole geometry. 

### 3.2. Discussion on Surface Roughness 

**Figure 11:**
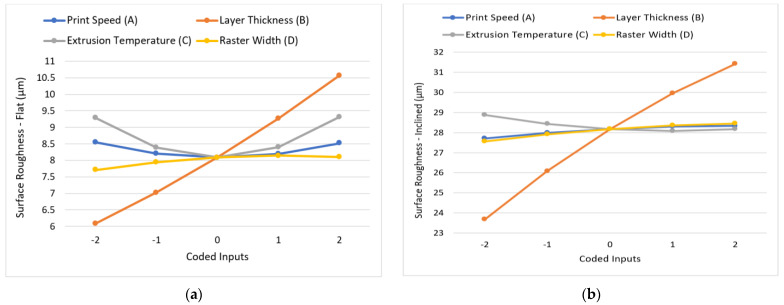

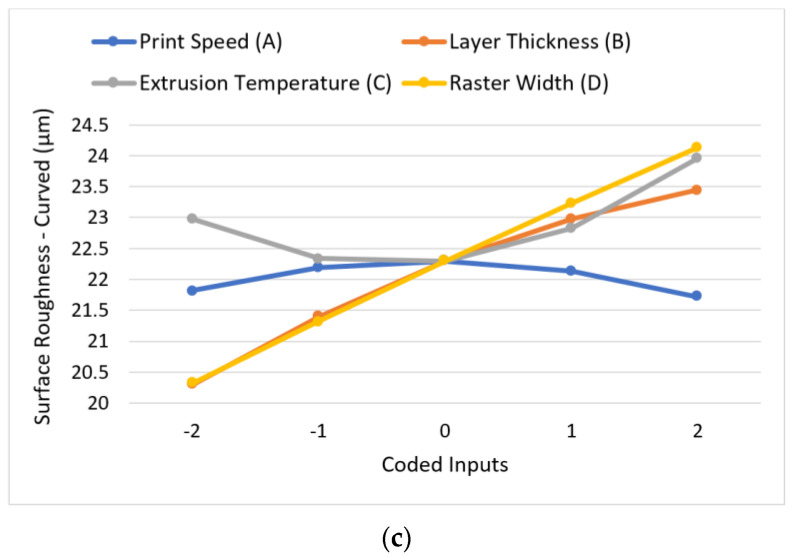
Effect of variation of input parameters on surface roughness: (**a**) flat; (**b**) inclined; (**c**) curved.

The graphs obtained for the variation in surface roughness with the varying printing parameters are shown in Figure 11a–c. For all the three surfaces considered, ANOVA returned layer thickness and raster width to be the significant parameters. It was observed from the plots that lower layer thickness values led to an improved surface finish, aligning with the majority of the literature reviewed [25,28]. This behaviour can be attributed to the reduced staircase effect owing to the precise layer tessellation under such premises. The trend for roughness magnitude variation was similar for raster width as well, with finer raster widths leading to an improved surface finish by allowing for the deposition of precise rasters. The precise rasters reduce the surface irregularities and roughness. 

The factors of print speed and extrusion temperature did not exercise a significant influence on the surface roughness, as also seen in Figure 11a–c, where the magnitude of surface roughness remained nearly the same while varying the said inputs. However, as per ANOVA, the combined interaction effect of print speed and extrusion temperature (AC) was observed to be a significant factor influencing the surface roughness of the flat profile. The quadratic term AC being significant highlights that the simultaneous effect of these parameters on surface roughness for the flat profile is significantly greater than the individual effects they exercise. 

**Figure 12:**
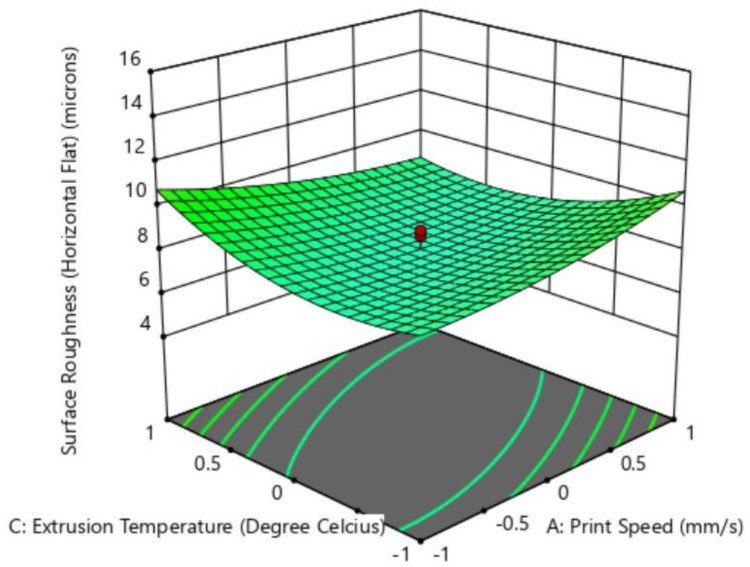
Combined effect of print speed and extrusion temperature on surface roughness for the flat profile.

Figure 12 gives the response plot for the combined interaction effect for AC. When extrusion temperature was fixed at its lowest value, i.e., −2 coded level (200 °C), and print speed was incremented, the surface roughness was observed to increase. This trend can be attributed to the vibrations induced in the nozzle system while depositing the thermoplastic material at high speeds. However, when the print speed was fixed at the −2 coded level (20 mm/s) and the extrusion temperature was increased, the part surface finish first improved then deteriorated. At lower printing temperatures, the material tended to stick to the nozzle during extrusion, leading to inconsistent deposition, which in turn was responsible for surface unevenness post-printing. In the same configuration, when temperature rises, surface finish improves slightly, however, beyond −1 coded level (i.e., 215 °C), the material fluidity increases, causing the material to spread in an unconstrained manner and solidifying unevenly, thereby introducing a poor surface finish. A similar trend was observed as the extrusion temperature was decreased while keeping the print speed fixed at a maximum coded level of +2 (80 mm/s).

A poor surface finish was observed when print speed was reduced while keeping the extrusion temperature constant at its maximum value. Although the lower print speed ensured a reduced dynamic drive effect and vibration, it gave more time for the highly fluid material (due to high extrusion temperature) to spread. Surface roughness was found to be the maximum (10.75 µm) when the extrusion temperature was at the highest coded level and the print speed was at its lowest value. 

Finally, a comparison of the surface roughness of the three different profiles revealed that the surface finish of the flat profile was considerably better than the other two profiles, with the inclined surface having the highest R_a_ value. The observations can be attributed to a pronounced staircase effect, especially for 3D-printed curvy surfaces. For the inclined surface profile, the issue was further compounded due to the nature of material deposition at the surface.

### 3.3. Validation of RSM, ANFIS and Hybrid RSM and NSGA-II Models

In the study, two predictive models, including RSM and ANFIS, were developed, and multi-objective optimization was performed using the hybrid RSM and NSGA-II method. As an attempt to validate the appropriacy of the developed models, the input parameter settings of the best run identified for performing PETG printing with the least dimensional deviations and high surface finish, as documented in Table 6, were supplied to the RSM and ANFIS models. The consequent responses, as predicted by the models, are documented in Table 8. The values returned by the predictive techniques were in close proximity to those suggested by the hybrid RSM-NSGA-II approach for the best run.

Moreover, for comparing the predictive performance of the developed ANFIS and RSM models, a comparison between the predicted values of the output responses from these models and the actual values after undergoing experimental validation was performed. The shifted parameter settings, based on the hybrid optimization approach for simultaneously optimizing all of the seven quality responses, as shown in Table 7, were chosen for the experimental run for validation. Three parts were printed and the output responses were measured using the predefined procedure. The mean values of the responses as measured along with the predicted responses from the RSM and ANFIS models are shown in Table 9. 

**Figure 13:**
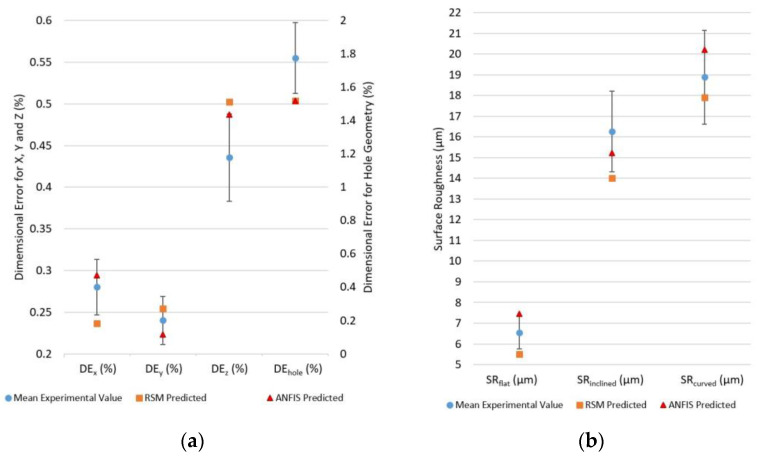
Error bars (uncertainty within ±12%) for experimental and predicted (RSM and ANFIS) values of (**a**) dimensional error response parameters and (**b**) surface roughness response parameters.

The experimental and the predicted value from the models were compared and the deviation of the predictions from the actual values were represented in the form of percentage error bars. Figure 13a,b highlight a higher deviation in the predicted values of dimensional error and surface roughness responses for the RSM design than the ANFIS predictive model. All the predicted values by the ANFIS model except for DE_hole_ were in ±12% of the mean experimental values; however, for RSM, most of the values deviated from the range of faults. As shown in Table 9, the average error in prediction from the ANFIS model was computed to be 9.33%, in contrast to an average error of 12.31% for RSM. Thus, ANFIS was found to have better predictive performance, which can be attributed to its superior knowledge learning capabilities. The limited accuracy of RSM arises due to the limitations in the polynomial estimation leading to a poor representation of the optimal parameters [14]. 

## 4. Conclusions

In the present study, an attempt was made to model the FDM process and conduct the multi-objective optimization of FDM-printed PETG parts. A series of experiments were performed using a four-factor central composite rotatable design to study and analyse the effects of print speed, layer thickness, extrusion temperature and raster width on dimensional error in different directions and for different surface roughness values of varied profiles of the built specimen. The statistical models for the responses were developed using RSM and their adequacy was verified using ANOVA. ANFIS models were also constructed for the prediction of output responses and multi-objective optimization was conducted using an integrated RSM and NSGA-II approach. 

ANOVA revealed layer thickness and raster width to be significant parameters for all the dimensional deviations. At lower values of layer thickness, the dimensional accuracy improved as the number of layers required for printing the part increased, further limiting the setup of non-uniform thermal gradients within the deposited material. Reduced raster width enabled the deposition of precise rasters and hence resulted in high dimensional accuracy in the built samples. The trend was attributed to two primary reasons. Firstly, fine raster widths ensured that less heat content entered the system environment, leading to reduced thermal stress accumulation at the interface during the solidification of the newly deposited material on the previous layer. Moreover, the radial expansion of polymer melt exiting the nozzle was also curtailed at a lower raster width, thereby reducing the dimensional errors. Print speed was also found to be a significant parameter for dimensional errors introduced along the length, width and thickness of the built parts. Lower print speed values led to improved dimensional accuracy owing to the reduced dynamic effect of the drive system. The effect of increased print speed was further pronounced in the Z direction owing to the drawing phenomenon, wherein the extruded material melt did not adhere to the previously deposited layers but moved with the extruder nozzle because of there being insufficient time available for the bonding to occur. A rise in extrusion temperature was found to enhance the material fluidity, causing excessive material spreading which led to marked dimensional errors along the X and Y directions. With the same process parameters settings, the dimensional error introduced in the hole geometry was found to be about four times higher than dimensional errors along the part’s length, width and thickness. This can be attributed to the tendency of the deposited material to spread, especially when unconstrained by surrounding geometry, as in the case of the hole.

Layer thickness and raster width were also significant parameters for the obtained surface roughness on the flat, inclined and curved surfaces. The direct trend plots showed surface finish to be better at smaller values of layer height and raster width due to the reduced staircase effect owing to the precise layer tessellation and allowing for the deposition of precise rasters, respectively. Moreover, the combined interaction effect of print speed and extrusion temperature was also found to exercise a significant influence on the surface roughness of the flat profile. The surface finish was found to be the poorest (10.75 µm) when the extrusion temperature was at the highest coded level and the print speed was at its lowest value. Furthermore, the surface finish obtained for the flat profile was best, followed by that of the curved and inclined profiles. The staircase effect and the nature of the material deposition are the chief contributors to this trend.

The hybrid RSM and NSGA-II multi-objective optimization approach was utilized to obtain the optimal printing settings for different premises. For the minimization of dimensional error, the optimal printing settings are a print speed of 65 mm/s, layer thickness of 0.2 mm, extrusion temperature of 215 °C and raster width of 0.2 mm. For printing PETG parts with the highest surface quality, the optimal settings are a print speed of 50 mm/s, layer thickness of 0.1 mm, extrusion temperature of 215 °C and raster width of 0.6 mm. Finally, for printing parts with the least dimensional errors and a high surface finish, the optimal settings as given by the approach are a print speed of 50 mm/s, layer thickness of 0.1 mm, extrusion temperature of 230 °C and raster width of 0.6 mm.

Upon validation with experimental runs, the predictive performance of the ANFIS model and RSM were compared, with the mean experimental values and the deviations being represented in the form of error bars. It was observed that, for the ANFIS models, all the predicted values except for DE_hole_ were in ±12% of the mean experimental values, but in the case of RSM, most of the values deviated from the range of faults. The mean error percentage for the predictions made by ANFIS was recorded to be 9.33%, which is lower in comparison to that obtained for RSM (12.31%). The better predictive performance of ANFIS can be attributed to its superior knowledge learning capabilities.

## Figures and Tables

**Table 1:** Working levels of input parameters.

S. No.	Input Parameters	Units	Coded Levels of Input Parameters				
			−2	−1	0	+1	+2
1	Print Speed (A)	mm/s	20	35	50	65	80
2	Layer Thickness (B)	mm	0.1	0.15	0.2	0.25	0.3
3	Extrusion Temperature (C)	°C	200	215	230	245	260
4	Raster Width (D)	mm	0.2	0.3	0.4	0.5	0.6

**Table 2:** Measured responses for dimensional accuracy for X, Y and Z axes and the hole geometry along with surface roughness responses for flat, inclined and curved surfaces.

Std	Run	Print Speed (mm/s) (A)	Layer Thickness (mm) (B)	Extrusion Temperature (°C) (C)	Raster Width (mm) (D)	Dimensional Error (%)				Surface Roughness (µm)		
						X	Y	Z	Hole	Flat	Inclined	Curved
24	1	0	0	0	2	0.8	0.387	0.684	1.975	7.659	29.37	26.98
4	2	1	1	−1	−1	0.613	0.516	0.338	1.925	10.938	31.686	23.53
20	3	0	2	0	0	0.787	0.493	0.853	2.3	13.25	33.97	25.297
16	4	1	1	1	1	1.24	0.427	1.04	2.35	10.957	30.928	26.672
1	5	−1	−1	−1	−1	0.427	0.067	0.4	1.775	6.695	22.891	20.046
22	6	0	0	2	0	0.953	0.464	0.373	2.59	12.807	28.915	29.576
26	7	0	0	0	0	0.827	0.427	0.338	2.14	7.638	27.934	24.105
14	8	1	−1	1	1	0.667	0.262	0.453	2.25	9.26	21.838	21.376
21	9	0	0	−2	0	0.64	0.28	0.453	2.125	12.584	31.222	24.885
7	10	−1	1	1	−1	0.56	0.16	0.373	1.89	15.78	28.087	20.305
18	11	2	0	0	0	0.973	0.433	0.453	2.375	9.62	28.617	19.778
28	12	0	0	0	0	0.813	0.507	0.347	2.525	8.685	27.170	23.793
11	13	−1	1	−1	1	0.48	0.245	0.56	2.175	10.48	32.59	24.111
30	14	0	0	0	0	0.867	0.4	0.453	2.14	8.935	28.554	21.911
29	15	0	0	0	0	0.733	0.373	0.32	2.125	7.671	29.970	22.265
3	16	−1	1	−1	−1	0.4	0.16	0.307	1.825	13.559	28.436	21.107
27	17	0	0	0	0	0.76	0.373	0.373	2.175	7.725	27.415	22.566
15	18	−1	1	1	1	0.72	0.36	0.48	2.22	15.255	32.896	25.422
2	19	1	−1	−1	−1	0.467	0.08	0.16	1.575	9.622	25.426	17.943
13	20	−1	−1	1	1	0.6	0.425	0.115	1.825	6.618	23.372	23.75
6	21	1	−1	1	−1	0.64	0.213	0.235	1.91	6.261	24.102	19.682
5	22	−1	−1	1	−1	0.413	0.107	0.258	1.6	6.141	24.469	23.052
12	23	1	1	−1	1	0.8	0.373	0.729	2.05	11.38	33.789	27.88
25	24	0	0	0	0	0.64	0.387	0.373	2.14	7.875	27.99	19.109
19	25	0	−2	0	0	0.453	0.147	0.293	2.01	4.357	18.399	16.357
10	26	1	−1	−1	1	0.613	0.2	0.507	2.125	11.745	22.445	22.596
8	27	1	1	1	−1	0.72	0.558	0.8	1.9	9.005	31.162	19.658
23	28	0	0	0	−2	0.467	0.126	0.435	1.4	6.589	26.606	17.517
9	29	−1	−1	−1	1	0.493	0.353	0.453	1.85	4.97	23.373	20.266
17	30	−2	0	0	0	0.613	0.293	0.267	2.261	9.619	27.484	21.066

**Table 3:** Analysis of variance for the quadratic models developed.

S. No.	Output Parameter	*p*-Value	R^2^	Adeq Precision	Adequacy of the Model
1.	Dimensional Error X	0.0001	0.8814	11.9315	Adequate
2.	Dimensional Error Y	<0.0001	0.9388	15.0776	Adequate
3.	Dimensional Error Z	<0.0001	0.9592	22.0652	Adequate
4.	Dimensional Error Hole	0.0034	0.8061	9.8613	Adequate
5.	Surface Roughness Flat	<0.0001	0.9872	32.2411	Adequate
6.	Surface Roughness Inclined	<0.0001	0.9732	25.9217	Adequate
7.	Surface Roughness Curved	0.0002	0.8756	10.3616	Adequate

**Table 4:** Factors significantly affecting output parameters.

Output Parameter	Significant Factors
Dimensional error X	A, B, C, D, B^2^, D^2^
Dimensional error Y	A, B, C, D, AB, AD, BD, B^2^, D^2^
Dimensional error Z	A, B, D, AB, AC, AD, BC, BD, CD, B^2^, D^2^
Dimensional error hole	B, D, D^2^
Surface Roughness Flat	B, D, AB, AC, AD, BC, BD, CD, A^2^, B^2^, C^2^
Surface Roughness Inclined	B, D, AD, BD, B^2^
Surface Roughness Curved	B, D, C^2^

**Table 5:** Average testing error for output responses with different membership functions.

S. No.	Membership Functions	RMSE of Dimensional Error				RMSE of Surface Roughness		
		X	Y	Z	Hole	Flat	Inclined	Curved
1	Triangular	0.12947	0.2583	**0.20134**	**0.32513**	**0.73702**	**0.82783**	**0.70272**
2	Trapezoid	**0.12841**	0.25678	0.2572	0.32513	0.73702	0.94533	0.70272
3	Gaussian	0.12896	**0.25582**	0.23218	0.33556	0.73702	1.0165	0.87578

**Table 6:** Parameter settings for optimization of quality responses as recommended by the hybrid RSM and NSGA II approach.

				Minimization of Dimensional Error						
Print Speed (A)	Layer Thickness (B)	Extrusion Temperature (C)	Raster Width (D)	Dimensional Error (%)						
				X		Y		Z		Hole
1.26	−0.47	−1.45	−1.97	0.34		0.09		0.05		1.14
				**Minimization of Surface Roughness**						
				**Surface Roughness (µm)**						
				**Flat**		**Inclined**		**Curved**		
−0.36	−1.99	−0.78	1.95	4.58		15.16		16.86		
				**Minimization of all Quality Responses**						
				**Dimensional Error (%)**				**Surface Roughness (µm)**		
				X	Y	Z	hole	Flat	Inclined	Curved
−0.17	−1.91	−0.39	1.56	0.3	0.28	0.5	1.67	5.07	16.04	17.65

**Table 7:** Shifted parameter settings for optimizing dimensional error and surface roughness.

Parameters Optimized	Print Speed (mm/s) (A)	Layer Thickness (mm) (B)	Extrusion Temperature (°C) (C)	Raster Width (mm) (D)
Dimensional Error	1	0	−1	−2
Surface Roughness	0	−2	−1	2
Dimensional error and Surface Roughness	0	−2	0	2

**Table 8:** Predicted responses for obtaining the best part quality.

Method	Dimensional Error (%)				Surface Roughness (µm)		
	X	Y	Z	Hole	Flat	Inclined	Curved
RSM-NSGA-II	0.31	0.278	0.5	1.67	5.07	16.04	17.65
RSM	0.641	0.36	0.37	2.08	6.33	23.48	21.08
ANFIS	0.60	0.07	0.46	2.14	7.39	17.3	21.2

**Table 9:** Comparison of part quality between experimental validation and model prediction.

Quality Response	Mean Experimental Value	RSM		ANFIS	
		Predicted	Error (%) in Prediction	Predicted	Error (%) in Prediction
${\mathrm{DE}}_{x}$ (%)	0.28	0.2367	15.46	0.294	5
${\mathrm{DE}}_{y}$ (%)	0.24	0.2542	5.92	0.223	7.08
${\mathrm{DE}}_{z}$ (%)	0.435556	0.5022	15.3	0.487	11.81
${\mathrm{DE}}_{\mathrm{hole}}\;$(%)	1.775	1.5182	14.47	1.52	14.37
${\mathrm{SR}}_{\mathrm{flat}}$ (µm)	6.558	5.5068	16.03	7.452	13.63
${\mathrm{SR}}_{\mathrm{inclined}}$ (µm)	16.252	13.9996	13.86	15.216	6.37
${\mathrm{SR}}_{\mathrm{curved}}$ (µm)	18.879	17.9076	5.15	20.21	7.05
Mean Error in Prediction (%)			12.31		9.33

## Data Availability

The recorded experimental data utilized in the study has been included in the article.
